# The Influence of Host Plant Extrafloral Nectaries on Multitrophic Interactions: An Experimental Investigation

**DOI:** 10.1371/journal.pone.0138157

**Published:** 2015-09-22

**Authors:** Suzanne Koptur, Ian M. Jones, Jorge E. Peña

**Affiliations:** 1 Department of Biological Sciences, Florida International University, Miami, Florida, United States of America; 2 Tropical Research and Education Center, University of Florida, Homestead, Florida, United States of America; Texas A&M University at Galveston, UNITED STATES

## Abstract

A field experiment was conducted with outplantings of the native perennial shrub *Senna mexicana* var. *chapmanii* in a semi-natural area adjacent to native pine rockland habitat in southern Florida. The presence of ants and the availability of extrafloral nectar were manipulated in a stratified random design. Insect communities were monitored and recorded over a period of six months with a view to addressing three main questions. Do ants provide biotic defense against key herbivores on *S*. *chapmanii*? Is the presence of ants on *S*. *chapmanii* mediated by EFN? Finally, are there ecological costs associated with the presence of ants on *S*. *chapmanii*, such as a reduction in alternative predator or parasitoid numbers? Herbivores on *S*. *chapmanii* included immature stages of three pierid butterflies, and adult weevils. Eight species of ants were associated with the plants, and other predators included spiders, ladybugs, wasps, and hemipterans. Parasitic, haemolymph-sucking midges (Ceratopogonidae) and parasitoid flies were also associated with the caterpillar herbivores, and possibly the extrafloral nectaries of the plants. The presence of ants did not appear to influence oviposition by butterflies, as numbers of lepidopterans of all developmental stages did not differ among treatments. Significantly more late instar caterpillars, however, were observed on plants with ants excluded, indicating that ants remove small caterpillars from plants. Substantially more alternative predators (spiders, ladybugs, and wasps) were observed on plants with ants excluded. Rates of parasitization did not differ among the treatments, but there were substantially fewer caterpillars succumbing to virus among those collected from control plants.

We provide a rare look at facultative ant-plant mutualisms in the context of the many other interactions with which they overlap. We conclude that ants provide some biotic defense against herbivores on *S*. *chapmanii*, and plants benefit overall from the presence of ants, despite negative impacts on non-ant predators.

## Introduction

Extrafloral nectaries (EFNs) have been reported in 93 plant families and 332 genera [1], and may be found on almost any vegetative or reproductive plant structure [1, 2, 3, 4]. While extrafloral nectar (EFN) may be consumed by a broad range of arthropods, its discovery by ants is thought to be of greatest importance to plant defense [1, 5, 6]. While defending their resource, many ant species show territorial aggressiveness towards, or even prey on other insects that they encounter [1].

Since Thomas Belt first hypothesized the mutualism between plants and defensive ants [7], many studies have supported ants as biotic defenders of plants [6, 8, 9, 10]. Ant-plants may be placed into two categories based on their defensive strategy. Myrmecophytic plants provide nesting sites and are permanently occupied by specialized ant species, while myrmecophylic plants provide unspecialized food rewards, most commonly extrafloral nectar (EFN) or honeydew (through associations with honeydew producing hemipterans), and foster only facultative interactions with ants. While the defensive role of ants on myrmecophytic plants is well supported [8, 9, 10], the defensive benefits of EFN attracted ants have been empirically demonstrated only relatively rarely [11, 12, 13, 14]. Indeed, several studies of EFN mediated ant-plant interactions have observed neutral or even negative effects on plant fitness [15, 16, 17]. In some cases the chemical composition of EFN appears to be tailored to attract defensive ants, and discourage exploiters [18]. Many facultative mutualisms between ants and EFN producing plants, however, offer low levels of species specificity and nectar is, therefore, available to be exploited by a host of arthropods which may confer no benefits to the plant (nectar thieves), and may even deter ants [19]. For example, 14 families of Diptera and 5 families of wasps have been observed at the EFNs of Lima beans alone [20]. The importance of EFN on the biology of non-ant consumers, however, has rarely been studied [21].

Ants, themselves, vary in their defensive qualities [9, 22, 23], and even the most effective ant bodyguards may not be exclusively beneficial for plants [24]. Mutualisms between plants and ants do not occur in isolation, but within a complex web of biotic interactions. In the cactus *Ferocactus wislizeni*, for example, plants defended by the most aggressive ants, *Solenopsis xyloni*, suffer reduced herbivory and produce more flowers. Those flowers, however, receive fewer and shorter visits from pollinators, deterred by the same ferocious ants [24]. Other studies have also observed that pollinators recognize the danger posed by ants [25, 26]. Assunção et al. [26] placed plastic ants on the petals of *Heteropterys pteropetala*. Flowers with plastic ants produced significantly less fruit than control flowers. Aggressive ants have also been observed to reduce the numbers of other beneficial insects, such as predators and parasitoids, on EFN bearing plants [6, 22]. In the EFN bearing tree, *Qualea multiflora*, both ants and spiders reduce herbivory rates, and an interaction effect has been observed whereby the best protected plants are those that harbor both ants and spiders. In many cases, however, ants outcompete spiders, with detrimental effects on plant defense [27].

Conflict between mutualistic guilds (bodyguards, pollinators, parasitoids) represents an important and understudied ecological cost of indirect plant defenses, and may be most prevalent in generalized systems where there is greater variation in partner quality and the relative importance of each mutualism. If we hope to understand the ecological role of EFN, along with the costs and benefits of ant-plant mutualisms, we must consider the whole network of interactions in which they exist. Ant-plant associations have been described as keystone interactions with the potential to dramatically alter the structure of arthropod communities [6]. The temporal dynamics of such mutualisms, however, and the balance of costs and benefits received by the plant, have rarely been studied in the context of the community [28, 29, 21].

*Senna* is a relatively large genus (300–350 species) of Caesalpinoid legumes, and one of the three richest genera in terms of the number of EFN bearing species, along with *Passiflora* and *Inga* [30]. The diversification of *Senna* has been attributed to the evolution of EFNs, facilitating interactions with opportunistic ants, and the exploitation of newly arising ecological niches [30]. Although the ecology of EFNs in *Senna* is not well understood, there have been a number of studies on EFN mediated interactions in *Senna* and closely related taxa.

In the present study we observed and recorded insect activity in *Senna mexicana* var. *chapmanii* (hereafter referred to as *Senna chapmanii*) over a period of six months, and compiled a large database of species occurring on our study plants. We examined the role of EFN in mediating ant-plant interactions in *S*. *chapmanii*, as well as the effects of ants on predator and herbivore numbers. Additionally, we investigated how the presence of ants affects the rate of parasitization in *S*. *chapmanii*’s key herbivores, the Sulphur butterflies. Ant protection of caterpillars against parasitoids has been observed several times through the experimental exclusion of ants [31, 32]. In one such exclusion experiment, parasitization by tachinid flies and braconid wasps, resulted in 78% mortality in *Hemiargus isola* (Lepidotera: Lycaenidae) larvae, almost twice that observed in the presence of tending ants [32].

In this study, 60 seedlings of *S*. *chapmanii* were planted in semi-natural growing conditions. The presence of ants and the availability of EFN were manipulated to test three major hypotheses. First, we predicted that ants on *S*. *chapmanii* would provide defense against herbivores such as pierid butterflies. Secondly, we hypothesized that the presence of ants would be mediated by the availability of EFN. Finally, we predicted that, despite their defensive benefits, the presence of ants would come at some ecological cost to *S*. *chapmanii* plants, specifically, a reduction in the numbers of other predatory insects, or the rate of herbivore parasitization.

## Methods

Seeds for plants cultivated for our experiment were collected from Camp Owaissa Bauer under Research Permit # 0021 to SK, Natural Areas Management Miami-Dade County Park and Recreation Department. The experiment was conducted on the grounds of the University of Florida's Tropical Research Experiment Center (UF-TREC: 25°30’27.52”N, 8°30’13.67”W; elevation 2.4 m) with permission as JEP was on the faculty and SK an official Research Associate of UF.

### Study site

The experiment was carried out at UF-TREC in Homestead, Florida. The regional climate is classified as subtropical, with average annual temperatures fluctuating between 3.2–24.8°C in January and 22.7–32.4°C in July. The mean annual precipitation is 1496 mm. The site elevation is close to sea level, and consists of flat calcareous limestone rocklands that have been rock-plowed for agriculture. We utilized a 2 acre restoration area within the site. The restoration area was a rockland hammock, previously overgrown with exotic pest plants, that had been mostly cleared of all vegetation except for some large native trees in the center and on a few edges. Within 5 m of the western edge there is a stand of pine rockland habitat, a protected natural area.

### Study species

*Senna* Miller (Fabaceae: Caesalpinoideae), formerly included in the genus *Cassia*, is a genus of 300–340 species, of which 80% occur in the New World [33]. *Senna* spp. are mostly woody perennials with parapinnate leaves, some bearing foliar nectaries between the lowest pair of opposite leaflets (varying among species) [34]. *Senna mexicana* (Jacq.) H.S. Irwin & Barneby var. *chapmanii* (Isely) H.S.Irwin & Barneby is a native species of southern Florida, and is state-listed as threatened (Atlas of Florida Plants), occurring only in Miami-Dade and Monroe counties, as well as in the Bahamas and Cuba. This species grows in pine rockland habitat and rockland hammock edges as an upright or sprawling subshrub, up to 1.2 m in height, spreading broader than tall. The bright, showy, yellow flowers (ca. 2 cm diameter) offer no nectar to floral visitors, and are usually visited by bees collecting pollen by ‘buzzing’ the anthers [35, 36]. Extrafloral nectaries, however, occur on the pedicels of flowers in the inflorescences, as well as throughout the foliage between basal leaflets.

### Experimental design

*Senna chapmanii* plants were grown in a greenhouse at Florida International University, from seed collected from the pine rockland at Camp Owaissa Bauer in Homestead (under Research Permit # 0021 to SK, Natural Areas Management Miami-Dade County Park and Recreation Department). After 3 months, sixty plants were transplanted into the experimental site on the grounds of the University of Florida Tropical Research Experiment Station, in an area of approximately 8000m^2^. Plants were installed in an evenly spaced array, over the open areas not occupied by dense vegetation. Each plant was at least 4m from its nearest neighbor. Treatments were assigned systematically to ensure even distribution of treatments across the site. Plants were mulched with wood chips, and watered for two months until they were established.

The experiment consisted of four treatments, in which the presence of ants and the availability of EFN were manipulated. The two independent variables were ants (present or excluded) and EFN (available or unavailable). The sixty plants were randomly assigned to one of four treatments as follows: 1) Ants removed, and a sticky resin (Tanglefoot™) applied to the base of branches to prevent the transit of ants and other crawling insects; this treatment is designated TF. 2) Ants present: ants removed manually to control for the effects of removal, such as shaking of branches, but no tanglefoot applied, so their return was not prevented; this is the control designated C. 3) Nail polish (Sally Hansen “Hard as Nails”) applied to all nectaries on the plant to reduce nectar available to all visitors; this treatment is designated NP. 4) Nail polish dabbed on the back of each leaf, to test for its potential repellent effects; this treatment is designated NPC. Nylon nail polish effectively seals the nectaries and eliminates nectar production [37, 14] without damaging plant tissues on this species and others with fairly sturdy leaves. The assigned treatment was applied to three branches of each plant, and reapplied weekly throughout the course of the experiment. The arthropod censuses and collections were made from the selected branches. Though originally there were 15 plants in each treatment, final sample sizes of plants monitored were TF = 12, C = 15, NP = 12, and NPC = 11.

Plants were transplanted in March 2003, the cooler dry season in south Florida, so irrigation was needed to aid their establishment until the summer rains came in late May. Plants were all similar in size initially, but some grew taller and others broader, depending on their genotype. We chose branches that were held upright and did not touch the ground, and trimmed overlapping branches to eliminate pathways for crawling insects to access treatment branches. Observations began in the fall rainy season, September 2003, and exclusion experiments started in October 2003. The experiment continued through February 2004, a period of six months, spanning both the wet and dry season (from December onward). In nature, plants flower and fruit year round, though flowering is greater from November through May (personal observations).

### Data collection

Throughout the field season a weekly census was conducted for each plant during the morning hours (dawn until mid-day), always with sunny or partly cloudy weather, during which insect numbers were counted, and their behaviors monitored, on the three target branches. Predatory arthropods such as ants, spiders, wasps, bugs, ladybugs and flies, were noted along with whether they visited nectaries, removed herbivores, or fed on other insects. Vouchers were collected where species determination was necessary. Herbivores on the plants were also recorded (leaf-chewing weevils, and larvae of pierid butterflies and tortricid moths), but only the larvae of pierid butterflies were intensively sampled. Numbers of small larvae (1^st^ and 2^nd^ instars) were recorded, while mature (3^rd^, 4^th^ and 5^th^ instar) caterpillars were recorded, collected, and reared to glean information on rates of parasitization and disease. The larvae of tortricid moths were sporadically collected and reared for information on their species identity and parasitization. For each type of arthropod we counted the number of individuals at each census, except for ants, flies, and weevils, where only the presence or absence of each species was noted.

Caterpillars were maintained individually in the laboratory, in 1-gallon plastic bags, and fed on leaves until they died or pupated. Caterpillars/Pupae were kept in the bags until the emergence of adult butterflies or, in the case of dead or morbid individuals, until the emergence of parasitoids (such individuals were placed in plastic Falcon ™ tubes with loosened caps). Emerging butterflies or parasitoids could then be identified.

For each pierid caterpillar collected, we recorded whether it survived to adulthood or died. For those caterpillars that died, it was determined if their death was a result of parasitization (where a parasitoid was seen to emerge) or from a virus. The most common parasitoid, a tachinid fly, usually emerged as a larva from the pierid butterfly chrysalis, and pupated in the container. We later found that some of these flies pupated within the chrysalis, and died while emerging from both pupae cases. Some fly larvae presumably died before emergence, and were not detected. By this stage specimens were often desiccated to such an extent as to prevent reliable analysis and, therefore, it was not always possible to determine the exact cause of death. In such cases we assigned the category “maybe parasitized”. Virus infected caterpillars exhibited discoloration and turned black shortly after death; virus infected pupae neither eclosed a butterfly nor a parasitoid, but turned either black or a mosaic of colors. Dead specimens (larvae and pupae, presumed to be killed by virus) collected over the study were later sent to L. Solter (Illinois Natural History Survey) for examination, to determine if there was evidence of virus or microsporidia. This turned out to be impossible to determine as our specimens, left in tubes at room temperature, had acquired fungi and other decomposers that interfered with her observations; no one common pathogenic agent could be identified from these specimens.

### Data analysis

We summed the occurrences of all types of insects on each plant (actual counts for most types; only presence/absence for ants, flies, and weevils) over the course of the experiment. We used sums rather than averages as it was unlikely that individual insects would be on the same plant (except for caterpillars, which were removed at the third instar and later for rearing). Where actual numbers were recorded, counts were compared among treatments using Univariate Analysis of Variance. Where only presence/absence was recorded, insect occurrence was compared using Contingency Tables and the Kruskal-Wallis test (SPSS version 11).

Analyses were performed with both actual data of occurrences of the various arthropod groups and square-root transformed data, which always gave the same results, so we report the results with the actual data. Some plants died during the course of the experiment, succumbing to fungal blight; data from all plants that lived for at least two months were included. Post-hoc tests for between treatment comparisons were either Tukeys HSD (for equal variance) or Dunnet’s C or Games-Howell (depending on the sample size). Caterpillar rearing data were analyzed using contingency tables.

## Results

In all the analyses we sought to detect effects of the treatments on the abundance of the various arthropods associated with *Senna mexicana* var. *chapmanii*. We will consider each of the groups in turn, starting with the ants, continuing with other potential predators, and then the herbivores.

### Ants

**Fig 1:**
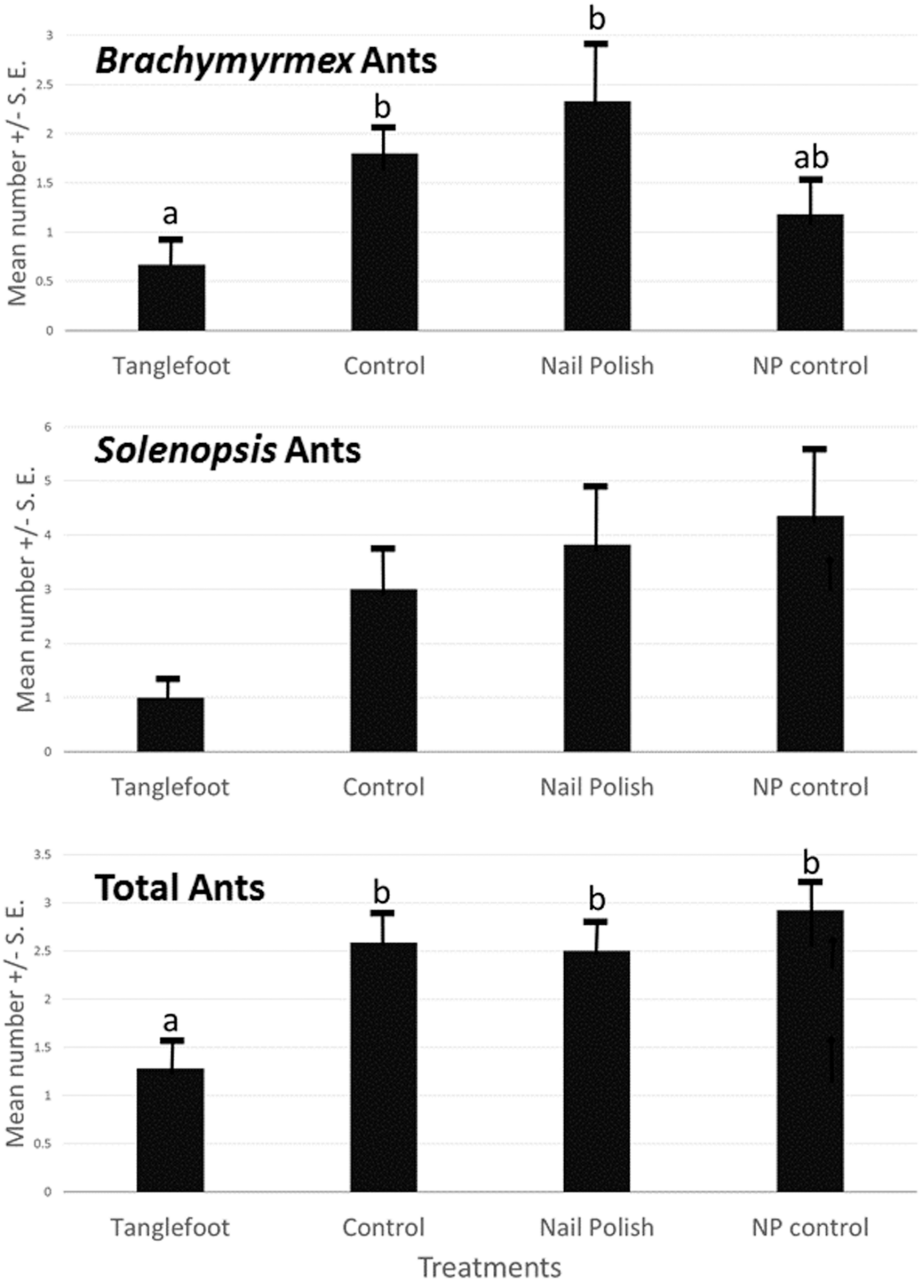
Ants on *Senna mexicana* var. *chapmanii* (experimental plantation) plants in Homestead, FL. Mean and standard error of numbers summed over the field season, over all plants, by treatment. Differences among treatments shown by Kruskal-Wallis for presence/absence data. Treatments abbreviated as C = control, TF = (tanglefoot) ant exclusion, NP = nail polish, NPC = nail polish control. Sample sizes of plants monitored were TF = 12, C = 15, NP = 12, and NPC = 11).

Eight species of ants were encountered on experimental plants (Table 1). Plants treated with Tanglefoot had the lowest numbers of ants of most species (lowest mean ranks in the Kruskal Wallis test), but only for one species of ant (*Brachymyrmex obscurior*; Kruskal Wallis X^2^_3_ = 9.2, p = .026) was the difference significant (Fig 1). Fire ants (*Solenopsis invicta*) did not differ significantly among treatments (X^2^_3_ = 2.822, p = 0.42) (Fig 1), and carpenter ants (*Camponotus*) were marginally significant when the three observed species were combined (X^2^_3_ = 7.23, p = 0.065). Overall, the desired effect of ant exclusion was obtained: all ant species combined were substantially less frequent on the Tanglefoot treated plants than on all the other treatments, which were not different from each other (Fig 1).

**Table 1:** Arthropods associated with experimental *Senna* plants.

Order, Family	Genus, Species, author	Nest location, ecological info, etc
Hymenoptera, Formicidae	Brachymyrmex obscurior* Wilson & Taylor	Ground (Tschinkel & Hess 1999)
	*Camponotus floridanus* Buckley	Decaying wood
	Camponotus rasilis* Wheeler	Decaying wood
	Camponotus sexguttatus* Fabricius	twigs
	*Odontomachus brunneus* Deyrup et al.	subterranean
	*Paratrechina longicornis* Latreille	Tree bark, rotten wood, stones, debris
	*Pseudomyrmex elongatus* Mayr	Dead twigs
	Pseudomyrmex gracilis* Fabricius	Twigs, branches
	Solenopsis invicta* Buren	ground
Hymenoptera, Halictidae	*Augochlorella* spp.	Flower visitor
	*Augochloropsis anonyma* Cockerell	Flower visitor
Hymenoptera, Apidae	*Melissodes communis* Cresson	Flower visitor
Hymenoptera, Vespidae	*Polistes major* (Beauvois) det. Deyrup 2003	Predator, make caterpillar meatballs
	*Pachodynerus nasidens* Latreille det Wiley 2005	Predator
Hemiptera, Alydidae	*Hyalymenus longispinus* Stål, 1870; det. D. Zisk 2005	ant-mimic nymphs—plant/seed feeders
Hemiptera, Reduviidae	*Zelus longipes* L.–D. Zisk 2005	predator, Assassin bug
Hemiptera, Pentatominae	*Euschistus* sp. det. D. Zisk 2005	predator
	*Loxa virescens* Amyot & Serville	predator
Hemiptera, Pyrrhocoridae	*Dysdercus mimulus* Hussey; det. J. Brambilia 2005	Plant feeder
Coleoptera, Cucurlionidae	*Diaprepes abbreviatus* Hussey; det. M.C. Thomas	Leaf feeder
	*Pachnaeus litus* Germar	Leaf feeder
Coleoptera, Coccinelidae	*Brachiacantha decora* Casey; det. MC Thomas	predator
	*Cycloneda sanguinea* Hussey	predator
	*Coelophora inaequalis* Fabricius det M.C. Thomas	predator
	*Diomus roseicollis* Mulsant	predator
	*Hippodamia convergens* Guérin-Méneville	predator
Arachnida, Thomisidae	*Misumenoides formosipes* Walckenaer 1837	predator

Table 1—Arthropods on *Senna mexicana* var. *chapmanii* plants in experimental plantation, Homestead, FL. Taxonomic information includes order, family, genus, species, and authority. Ant (Formicidae) species considered exotic are indicated with *. Brief ecological information is also provided for each documented association.

Of the 60 plants in the study, 18 were colonized by fire ant nests at their base during the course of the experiment: 9 of the NPC plants; 5 of the NP plants; and 2 each of the TF and C plants. During the course of the experiment, some of the plants died, succumbing to a type of blight, wilting one week, dead the next week. One or two of each group was lost in this way, reducing the sample size by the end of the study. Only one of the plants with a nest at its base died.

### Other Predators

**Fig 2:**
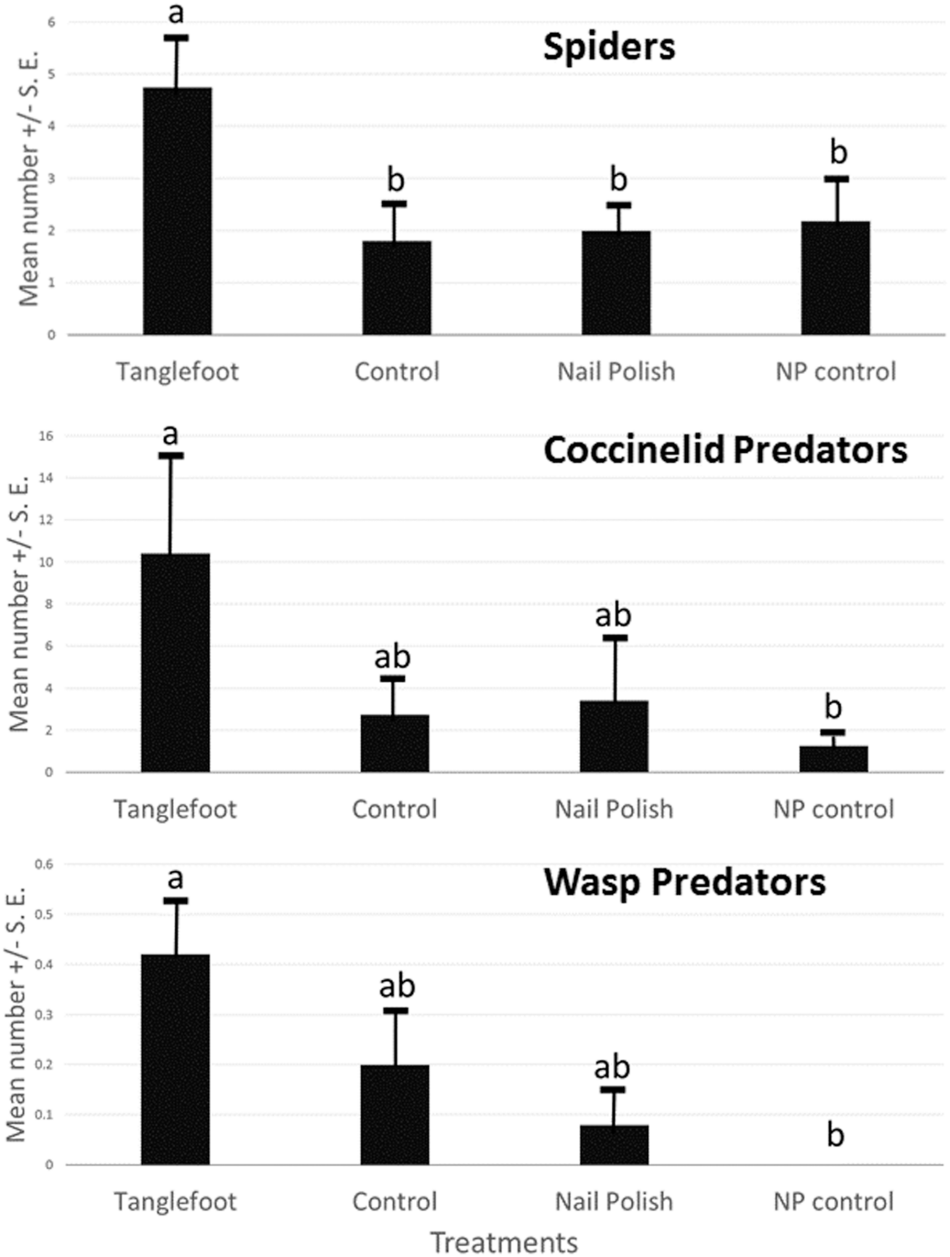
Other potential predators on *Senna mexicana* var. *chapmanii* (experimental plantation) plants in Homestead, FL. Mean and standard error of numbers summed over the field season, over all plants, by treatment. Differences among treatments shown by ANOVA for count data. Treatments and sample sizes the same as in Fig 1.

Predators of several orders (Hymenoptera, Hemiptera, Coleoptera) and many families were recorded on the *Senna chapmanii* plants in this experiment (Table 1; Fig 2). Several non-ant predators were substantially more abundant on plants with ants excluded: ladybugs (Cocinellidae) (F_3,47_ = 3.4, p = 0.025), spiders (Thomisidae) (F_3,47_ = 2.998, p = 0.040), and wasps (Vespidae) showed statistically significant differences (F_3,47_ = 2.82, p = 0.049) among treatments and were most abundant on Tanglefoot treated plants (Fig 2). Hemiptera predators (F_3,47_ = 1.56, p = 0.212) and sucking flies (Ceratopogonidae) (F_3,47_ = 1.999, p = 0.127) did not differ significantly among treatments.

### Herbivores

**Fig 3:**
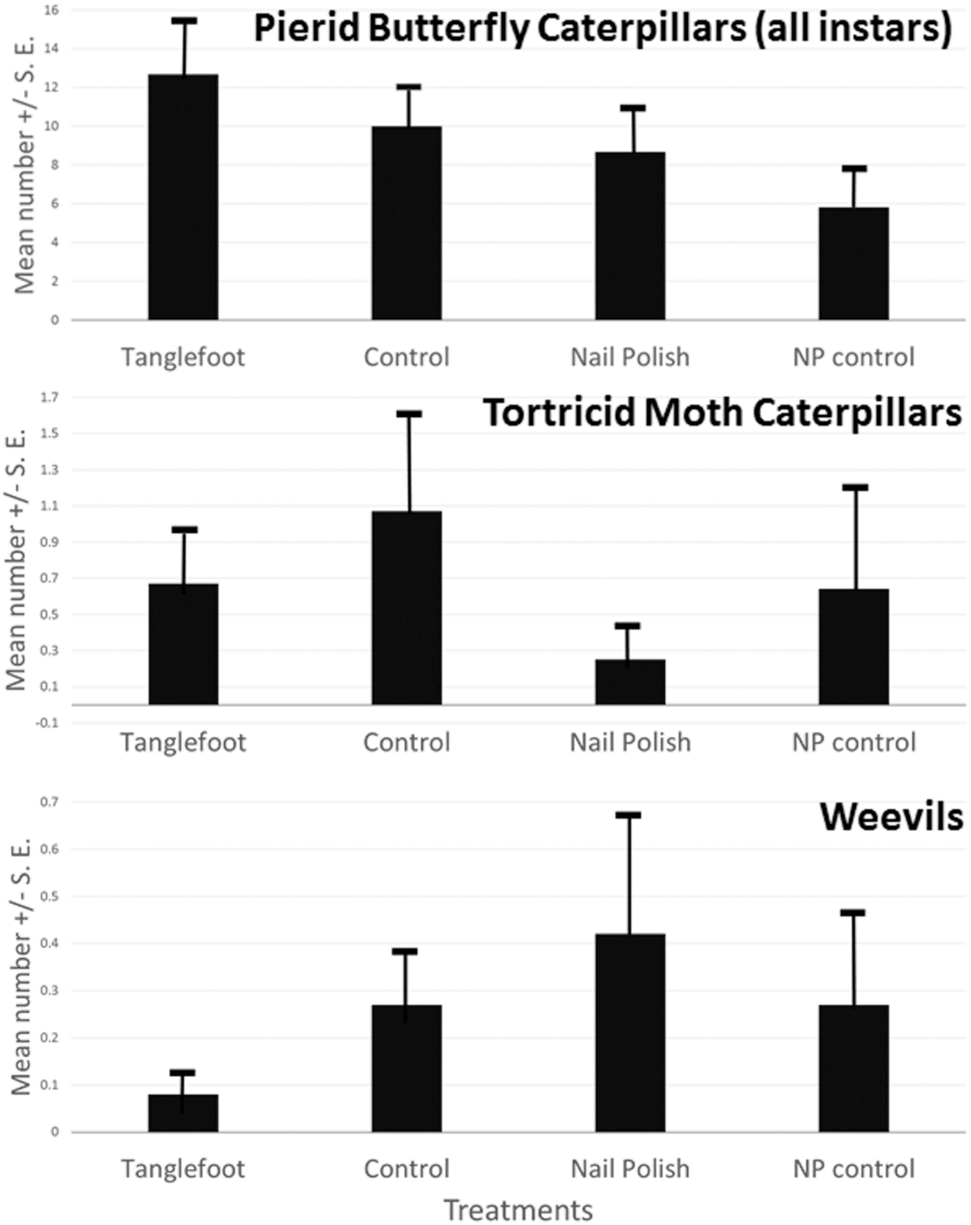
Herbivores on *Senna mexicana* var. *chapmanii* (experimental plantation) plants in Homestead, FL. Mean and standard error of numbers summed over the field season, over all plants, by treatment. Differences among treatments shown by ANOVA for count data. Treatments and sample sizes the same as in Figs 1 and 2.

Three pierid butterfly species occurred on *Senna mexicana* var. *chapmanii*: *Abaeis nicippe* (Cramer) (the sleepy orange), *Phoebis philea* (L.) (the orange-barred sulfur), and *Phoebis sennae* (L.) (the cloudless sulfur). *Abaeis nicippe* were the most numerous, followed by *P*. *sennae*, while *P*. *philea* were relatively rare (Fig 3). *Abaeis nicippe* larvae consumed only foliage, whereas the larvae of both *Phoebis* species consumed either leaves or flowers. *Phoebis* larvae had coloration reflecting the color of the plant organ consumed. Comparing the number of caterpillars collected at 3^rd^ instar and larger for rearing, we can see that for both *Phoebis* species, more were found on plants with ants excluded (*P*. *philea* X^2^_3_ = 141.24; p < 0.005; *P*. *sennae* X^2^_3_ = 78.25; p < 0.005), and this difference was significant also for comparing only ant exclusion (TF treatment) with the control for both *Phoebis* species as well (*P*. *philea* X^2^ = 17.82; p < 0.005; *P*. *sennae* X^2^_3_ = 16.6; p < 0.005. This difference between ant-excluded and control was not evident, however, for *Abaeis nicippe* (X^2^ = 0.08; p > 0.5), though overall there was a significant effect of treatment (X^2^_3_ = 107.22; p < 0.005). When caterpillars of all species collected for rearing were combined, there was an even more dramatic difference among treatments (X^2^_3_ = 195.6, p < 0.005) with many more caterpillars discovered, collected, and reared from plants with ants excluded versus control plants (X^2^ = 14.0; p < 0.005).

**Fig 4:**
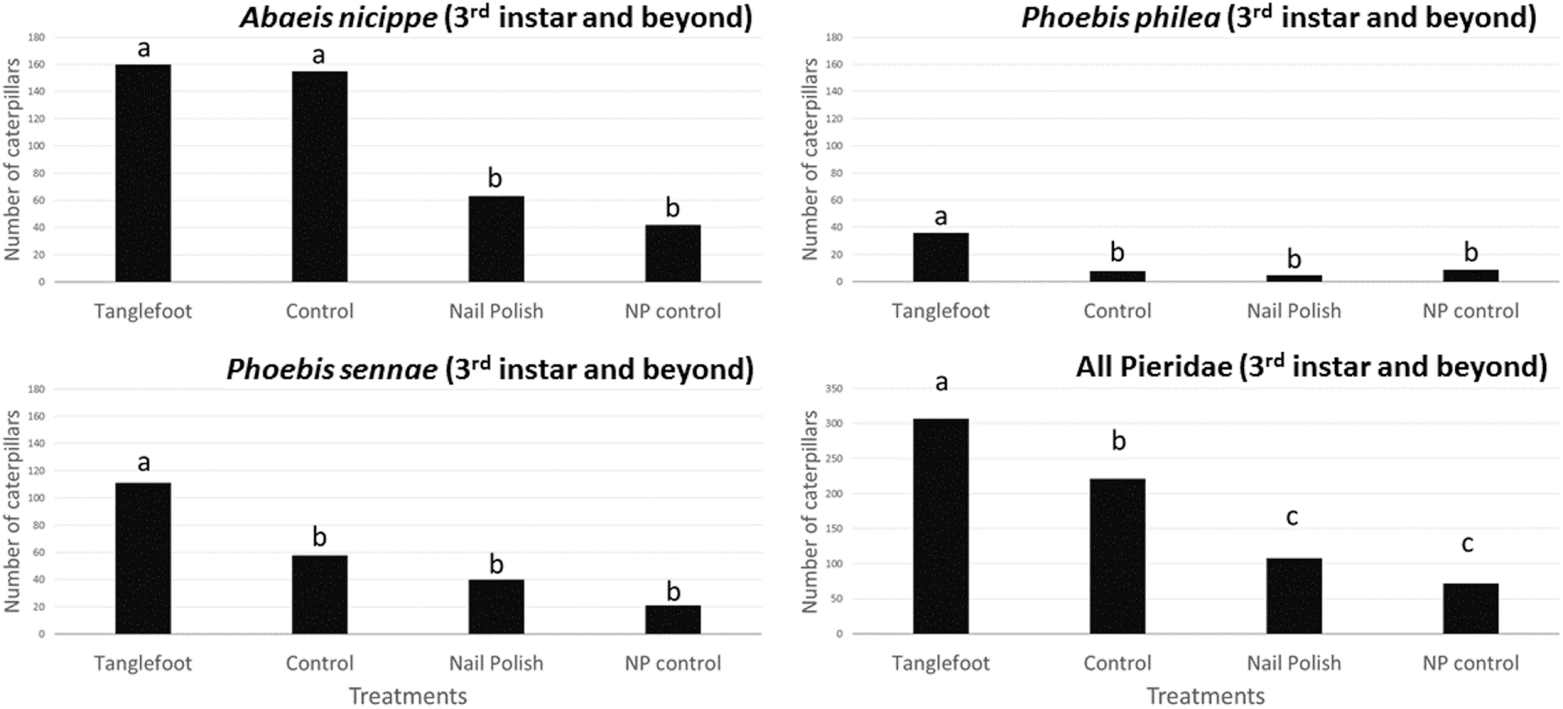
Pieridae (3^rd^ instar or higher) collected from field-grown *Senna mexicana var*. *chapmanii*, and brought to lab for rearing. Individuals, summed over the field season over all plants, are separated by species and treatment. n = number of plants monitored. Treatments and sample sizes of plants monitored the same as in Figs 1, 2 and 3. Pearson X^2^_3_ = 25.5, p < 0.0001.

Although all caterpillars were somewhat more numerous on plants with ants excluded (especially *Phoebis sennae*), the overall number of Pieridae encountered in weekly censuses (all species combined, and all stages from early through late instars), did not differ significantly among plants in different treatments (F_3,47_ = 1.288, p = 0.29) (Fig 3). Numbers of caterpillars collected and reared in the lab (3^rd^ instar and greater), however, differed significantly among treatments (Pearson X^2^_3_ = 34.997, p < .0001), with greater numbers found on plants from which ants were excluded (the TF treatment; Fig 4).

Several species of tortricid caterpillars (subfamily Phyticinae) were recorded, collected, and reared, and their numbers did not differ significantly among treatments (F_3,47_ = 0.591; p = 0.624; results not shown). Leaf-chewing weevils were also encountered on experimental plants, including *Diaprepes abbreviatus* and *Pachnaeus litus* (Coleoptera: Curculionidae). Their numbers did not differ significantly among treatments.

### Parasitoids

Two species of tachinid flies were reared as parasitoids from the two most common sulfur butterfly caterpillars, *A*. *nicippe* and *P*. *sennae*. One species was positively identified as *Lespesia parviteres* (Aldrich & Webber), while the other, a species of *Hyphantrophaga* (possibly *H*. *sellersi* (Sabrosky), could not be identified beyond the genus due to confusion in this taxon. Though the same flies reared as parasitoids were also observed several times in the field on plants and on researchers, their numbers were not great enough to warrant analysis. Small flies observed sucking the haemolymph of sulfur caterpillars were collected and determined by William Grogan to be *Forcipomyia* (*Microhelea*) *eriophora* (Williston) (Ceratopogonideae), a species recently observed in the Florida Keys feeding on the Florida leafwing butterfly [38]. Most of the caterpillars found with the sucking flies died before pupation [14], perhaps from a virus transmitted by the flies.

Only one wasp (Ichneumonidae) parasitoid individual was reared from the sulfur caterpillars, but this species was never observed in the field, though several other small wasps that might be parasitoids were observed on plants and at nectaries.

### Parasitization and caterpillar success

**Fig 5:**
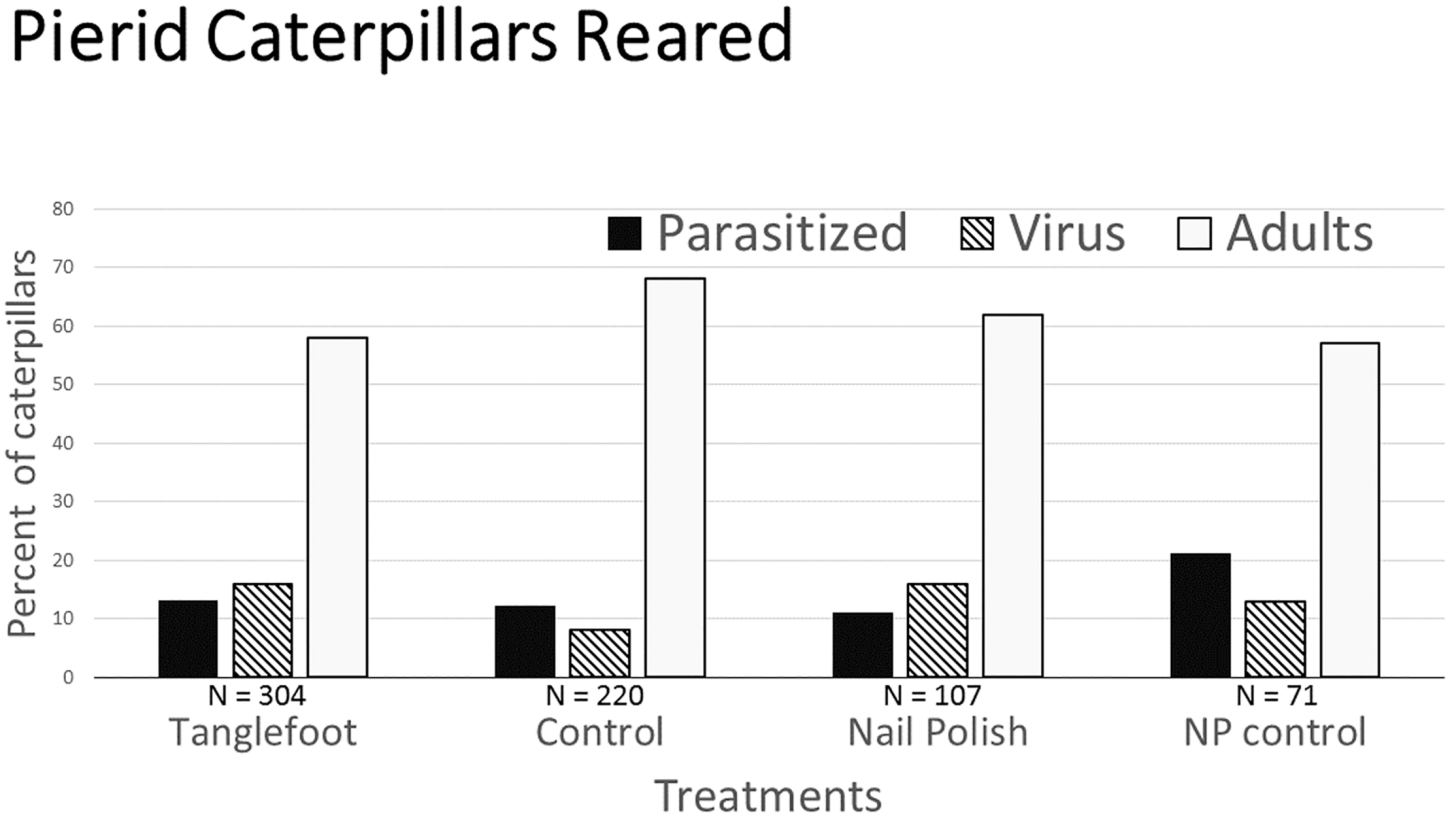
Rates of parasitization, virus death, and adulthood reached by caterpillars by treatment. Sample size is the number of caterpillars collected from plants (3^rd^ instar and beyond) and reared from plants in each treatment group. Parasitized counts include both definitely and “maybe parasitized” individuals.

Parasitization rate determined from caterpillars collected was 13% over all treatments. The rate did not differ significantly among treatments (Pearson X^2^_3_ = 4.35, p = 0.226; Fig 5).

Viruses were implicated in the death of many caterpillars, despite hygienic rearing protocol. Over all treatments, 13% of the individuals reared apparently succumbed to virus; this differed significantly among treatments, with caterpillars from Control plants faring substantially better than those from all the other treatments (Pearson X^2^_3_ = 8.75, p = 0.033; Fig 5).

Caterpillar success rate in becoming an adult butterfly was 61% over all treatments, and though not significant (Pearson X^2^_3_ = 7.38, p = 0.06; Fig 5), the best survival was seen in the caterpillars from Control plants (69% vs. 57–58% in all other groups).

## Discussion

Extrafloral nectaries are a way of plants attracting “pugnacious bodyguards” [3] as they subsidize the diet of carnivorous insects [39], forming the basis for protective mutualisms. The outcome of food-for-protection mutualisms between ants and plants are difficult to predict, however, as they don’t occur in isolation, but within a complex web of biotic interactions. By manipulating the presence of ants, and the availability of EFN, we cast some light on the many interactions mediated by EFN, and the role of ants as a biotic defense in *S*. *chapmanii*.

**Fig 6:**
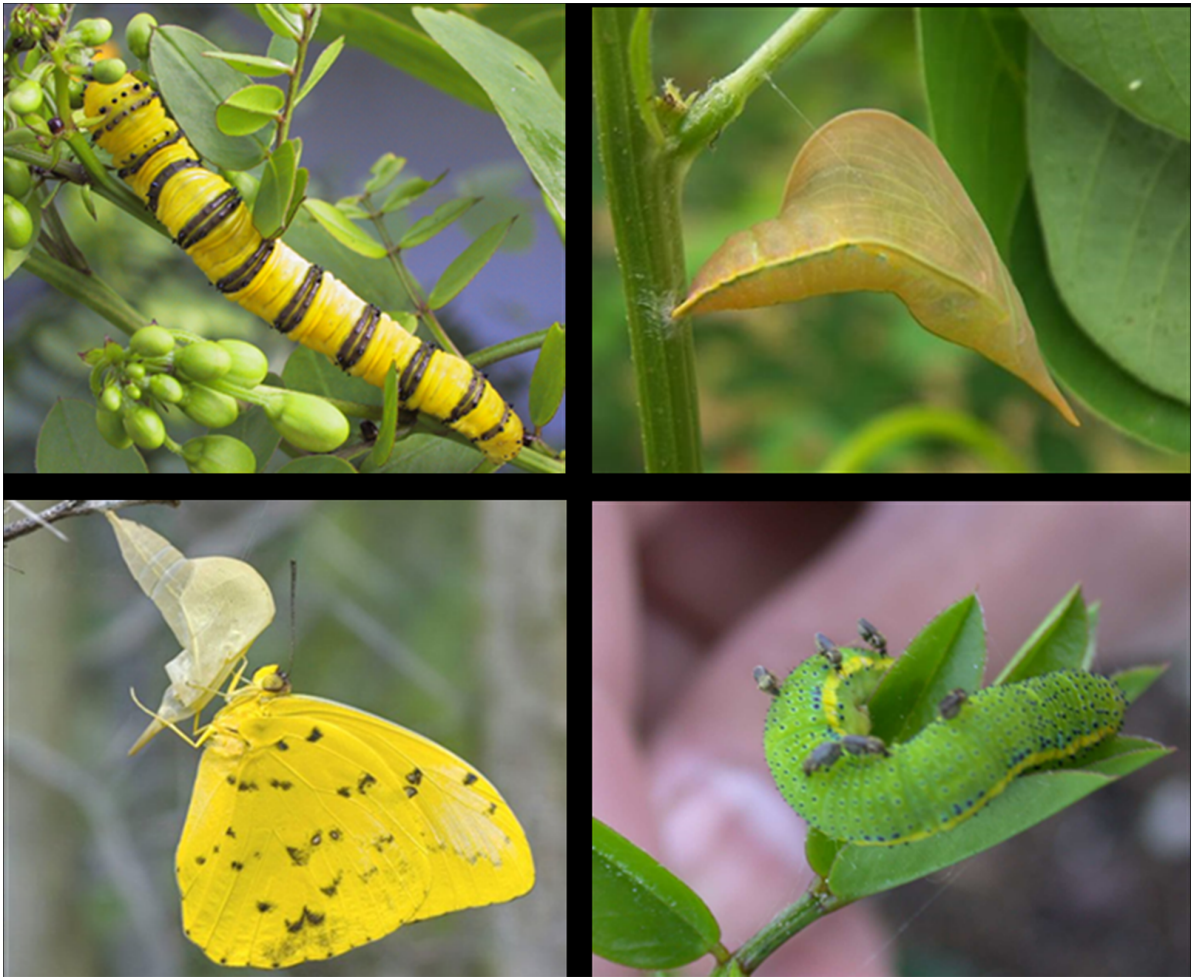
Predators on *Senna chapmannii* plants—upper left, *Polistes major* wasp with *Phoebis philea* caterpillar; upper right, *Polistes* wasp damage to *Phoebis sennae* chrysalis; lower right, coccinelid *Brachiacantha decora* adult at extrafloral nectary; lower left, thomisid spider *Misumenoides formosipes* ready for prey.

Although the presence of ants has been shown to deter oviposition by lepidopteran herbivores in several studies [4, 11, 40], our results suggest this is not the case in *S*. *chapmanii*, as overall numbers of pierid caterpillars were similar among treatments. Late instar pierid caterpillars were significantly less abundant in the presence of ants, however, indicating that ants defend *S*. *chapmanii* plants by removing these key herbivores (Fig 6). A very similar relationship between ants and pierid herbivores has been observed in a closely related species, *Senna occidentalis*. Fleet and Young [41] observed that oviposition by two Sulphur butterflies was not deterred by defensive ants, but that the survival rates of eggs and larvae were reduced. Several other studies have reported reduced levels of caterpillar infestation in the presence of ants [42, 43]. Sendoya and Oliveira [43] studied these effects at the habitat level (the Brazilian cerrado), and found that rates of caterpillar infestation were influenced by local variations in ant numbers and species, as well as the preference of those ant species for plants producing EFN.

We observed no differences in the incidence of tortricid caterpillars among treatments, and this was to be expected, as these are concealed feeders and are less likely to be affected by surface patrolling ants. Indeed, the presence of ants may even benefit some concealed feeders by deterring their enemies [44, 45]. Unfortunately, too few Tortricidae were reared in this study to determine if that is the case in this system.

The role of ants as plant defenders is well supported, but mainly in myrmecophytic plants that provide both food and shelter to their ant partners, and engage in specialized obligate mutualisms (for example, [8]). Senna and relatives, however, represent a large group in which the ecology of EFN has been relatively well studied, and several species have been observed to benefit from facultative relationships with ants. *Senna occidentalis* in Texas receives protection from fire ants against two of the same sulfur butterflies as in this study, resulting in greater plant height, number of leaves, and reproductive fitness [41]. In the Brazilian cerrado, *Chamaecrista debilis* nectaries are visited by ants that decrease herbivory and increase fruit set [46]. The nectaries of *Cassia fasiculata* have also been shown to support protective ants that reduce herbivore damage [47], and increase plant fecundity [48]. Boecklen [49], however, excluded ants from the same species using two methods (tanglefoot and excising nectaries, alone and in combination), concluding that the presence of ants was of no benefit to plants as treatment plants produced as many fruits as did control plants. It remains to be seen in our system if ants visiting nectaries enhance plant reproduction.

The application of Tanglefoot™ was effective in excluding ants from treatment branches over the course of the study. Reducing the availability of EFN using nail polish, however, did not significantly affect ant activity on the study branches. This was a surprising result, as many studies have linked ant activity with the availability of EFN [50, 20, 51]. Indeed, Baker-Meio and Marquis [52] studied several varieties of *Chamaecrista desvauxii* and found that those varieties with larger nectaries produced more nectar and attracted more ants. During our study, EFNs in the inflorescences of flowering individuals may have augmented ant activity on nail polish treated branches, as they were not always covered with nail polish. Additionally, on large study plants, only the experimental branches were treated, so the availability of EFN on non-test branches likely accounts for many of the ants attracted to these plants.

**Fig 7:**
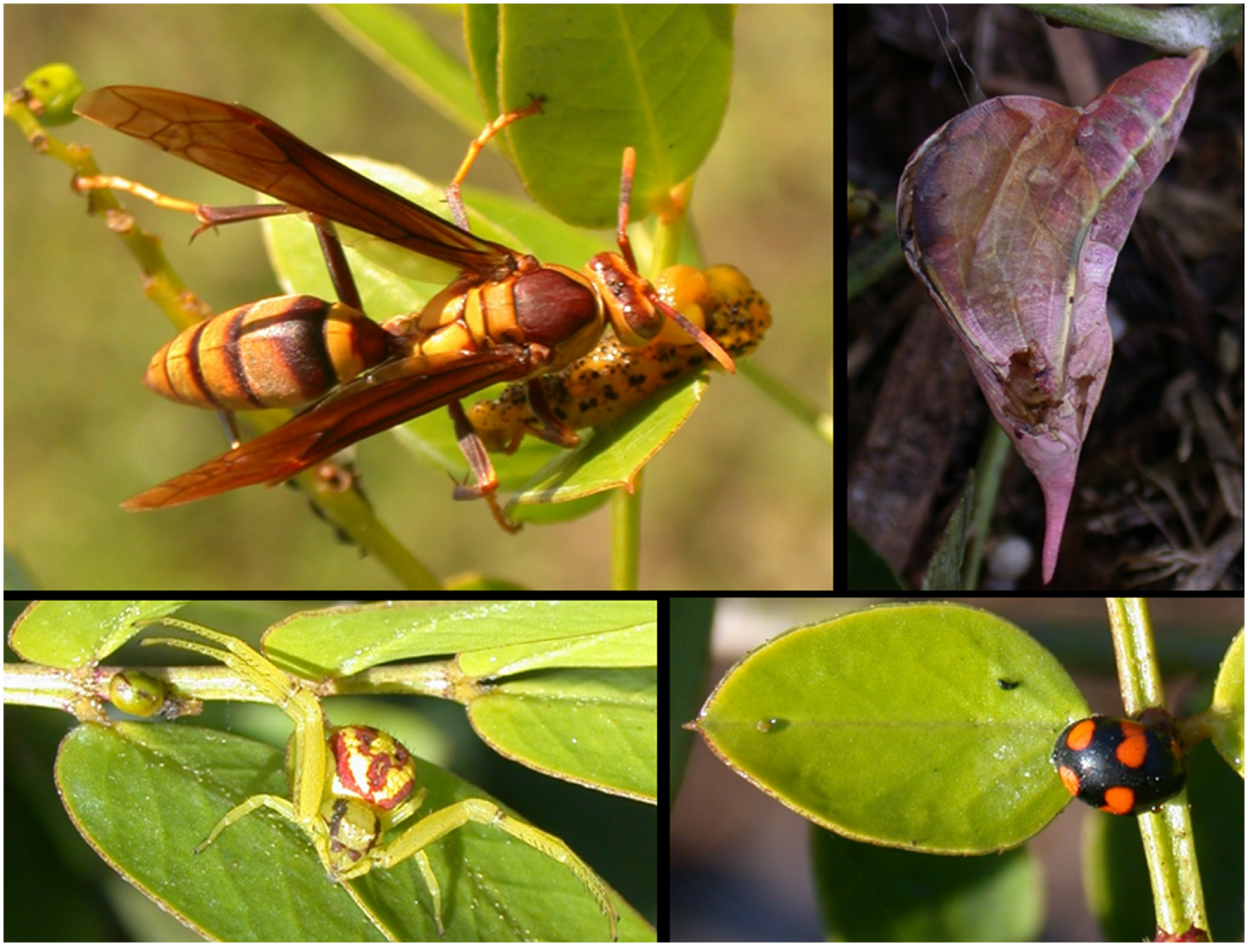
Some players in the tritrophic system—upper left, caterpillar of the orange-barred sulfur butterfly, *Phoebis philea*, on *Senna chapmanii*; upper right, pupa (chrysalis) of *P*. *sennae*; lower left, adult *P*. *sennae*; lower right: caterpillar studded with sucking flies (virus transmitters?). When viruses are involved, the pupae do not hatch, but instead turn various colors.

Our results demonstrate that when ants are excluded from nectaries in this study system, the numbers of other predators are significantly increased (Fig 7). The deterrence of other predators represents an important ecological cost of ant-plant interactions. Torres-Hernandez et al. [22] also found that predator numbers on *Turnera ulmifolia* were increased when ants were excluded, and that these predators provided better defense against herbivores than some ant species. Spiders have been shown to enhance seed set of *Chamaecrista nictitans* host plants with extrafloral nectaries [53], even when ants are ineffective at repelling some herbivores [45]. Vespid wasps visit extrafloral nectaries and are voracious predators (and we observed several making “caterpillar meatballs” during the course of the study). Wasps attracted to the extrafloral nectaries of *Turnera ulmifolia* have been shown to positively affect plant reproductive output [54]. Coccinellidae larvae and adults have been observed to visit extrafloral nectaries ([55], and the present study) and consume a variety of arthropods. Feeding on nectar and honeydew has even been shown to enhance development and survival these omnivorous “predators” [56].

In this study, neither the presence of ants, nor the availability of EFN, affected the rates of parasitization in pierid caterpillars. Although several studies have observed ants defending caterpillars from parasitoids [31, 32], these studies involved ant-tended lepidopteran species that are known to provide ants with sugary resources in return for defense. It has been observed [James Spencer, personal communication] that sulphur caterpillars at times produce tiny droplets on the tips of setae covering their bodies, but the potential role of this liquid as an ant reward, or ant-deterrent, has not yet been investigated. We have never observed caterpillar-tending behavior by ants, but the droplets may play a role in caterpillar protection (presently under investigation).

A surprising observation was that on control plants, where ant activity was high, fewer caterpillars died from viruses. We also observed that caterpillars collected from these plants had a higher rate of successful emergence as adults, although the result was not quite significant in this case. A variety of predators and parasitoids (Coccinellid larvae, tachinid flies, and parasitic wasps) have been shown to carry viable nuclear polyhedrosis virus [57]. It is possible; therefore, that the deterrence of predators by ants may explain the low rates of viral infection seen in caterpillars collected from control plants. An alternative explanation is that ants may disproportionally predate upon virus infected caterpillars, leading to lower rates of virus infection in reared caterpillars. While we view this explanation as unlikely, future work could determine if virus infected caterpillars are more or less vulnerable to predation.

An unanticipated dimension of the study was the discovery of caterpillar-sucking midges that might have a role in spreading virus and thereby controlling caterpillars. Ceratopogonidae midges (*Forcipomyia* (*Microhelea*) *eriophora*) were first observed feeding on *P*. *sennae* during this study, and the phenomenon was later observed several times in natural settings. The parasitized larvae were collected, and died at higher rates in captivity than is expected in rearings of this species [14]. It might be that these Ceratopogonidae midges are not as likely to suck caterpillars in the presence of ants, but more work is needed to test this, as well as the virus transmission hypothesis [14].

Like many EFN producing plants, *S*. *chapmanii* appears to engage in non-specialist facultative interactions with ants. In this study alone, the EFNs of *S*. *chapmanii* were regularly visited by eight species of ants. In addition to attracting small numbers of workers to defend against herbivores, some EFN producing plants may also attract ants to nest beneath the plants, increasing the reliability of defense and potentially providing additional nutrients in the soil [58, 59]. In this experiment, of the thirty plants with available EFNs, eleven had fire ant nests at their base. Study plants were mulched to prevent the growth of weeds that might function as ‘ant-bridges’, and this mulch may have contributed to the attraction of nesting ants, however, this unexpectedly high occurrence of nests surely warrants further investigation. Indeed, the outcomes of ant-plant mutualisms must ultimately depend more on the dynamics of colonies, than the behavior of individual workers, and we are not the first to suggest that future work might use colonies, rather than workers, as the unit of study [60].

We have previously shown that *S*. *chapmanii* plants produce more EFN in response to leaf damage, and that the same leaf damage elicits increased ant activity on the plants [51]. Although not comprehensive, the present study provides a record of many insects found on *S*. *chapmanii*, and represents a rare effort to describe EFN mediated ant-plant interactions in the context of the many other interactions around which they occur. We provide strong evidence that ants remove key herbivores from *S*. *chapmanii*, and future work should focus on how ants affect plant reproductive fitness in this system.
