# Correction: The Influence of Host Plant Extrafloral Nectaries on Multitrophic Interactions: An Experimental Investigation

**DOI:** 10.1371/journal.pone.0202836

**Published:** 2018-08-23

**Authors:** Suzanne Koptur, Ian M. Jones, Jorge E. Peña

The captions for Figs 6 and 7 are incorrectly switched. The images appear in the correct order. Please see the corrected captions here.

**Fig 6:**
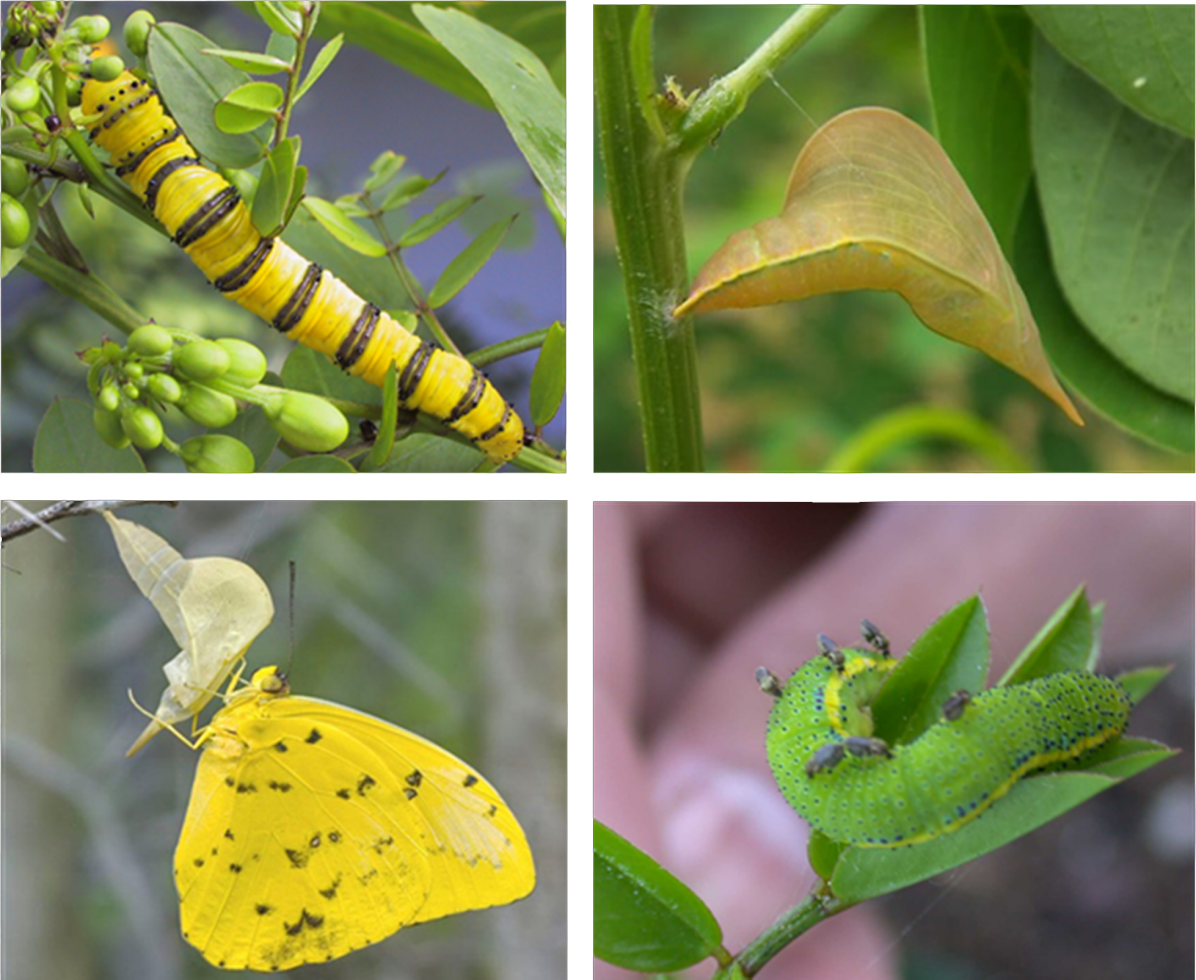
Some players in the tritrophic system—upper left, caterpillar of the orange-barred sulfur butterfly, *Phoebis philea*, on *Senna chapmanii*; upper right, pupa (chrysalis) of P. sennae; lower left, adult *P*. *sennae*; lower right: caterpillar studded with sucking flies (virus transmitters?). When viruses are involved, the pupae do not hatch, but instead turn various colors.

**Fig 7:**
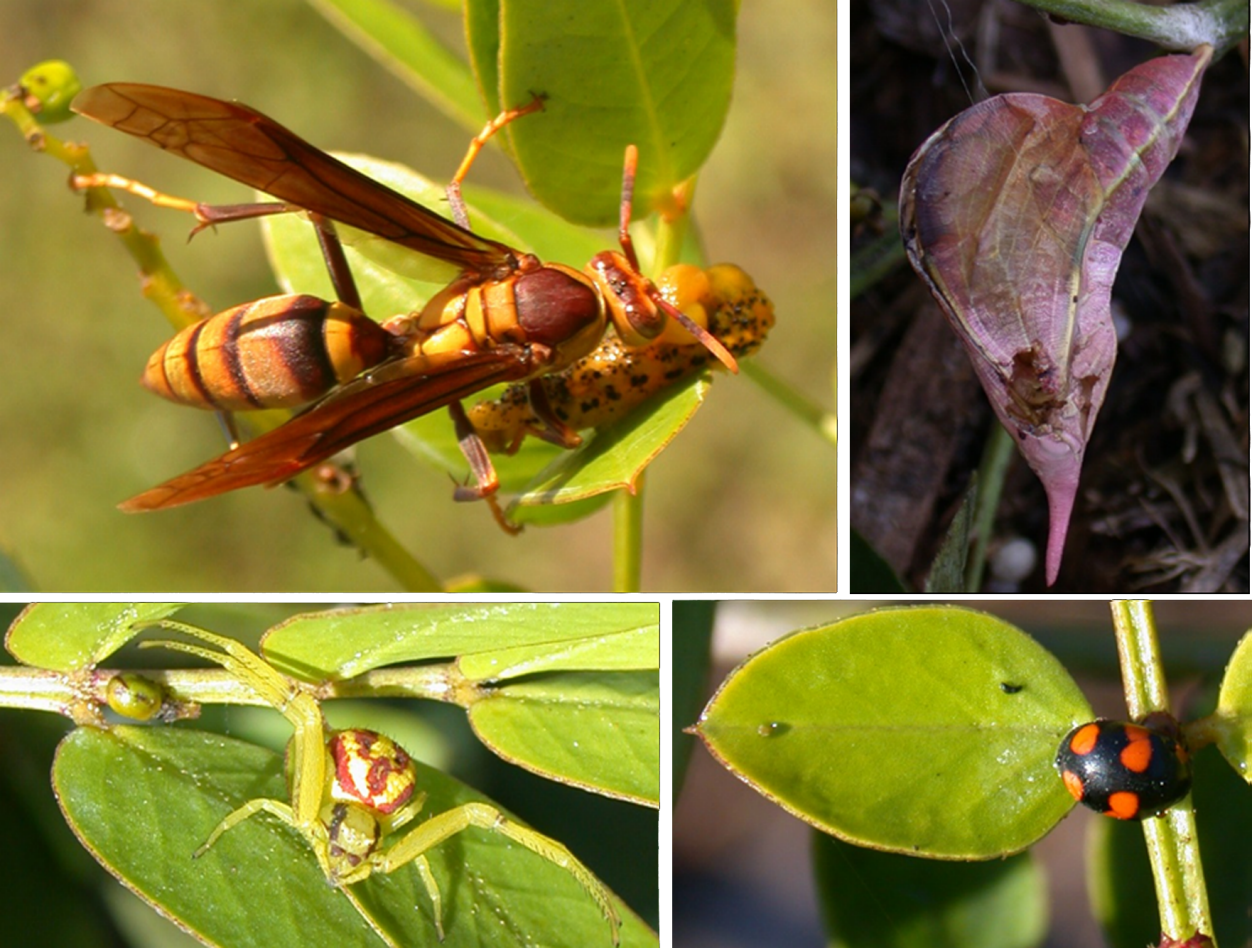
Predators on *Senna chapmannii* plants—upper left, *Polistes major* wasp with *Phoebis philea* caterpillar; upper right, *Polistes* wasp damage to *Phoebis sennae* chrysalis; lower right, coccinelid *Brachiacantha decora* adult at extrafloral nectary; lower left, thomisid spider *Misumenoides formosipes* ready for prey.
